# Supplementary figures and images for: Co-production of knowledge as part of a OneHealth approach to better control zoonotic diseases
Source: PLOS Glob Public Health. 2022 Mar 24;2(3):e0000075. doi: 10.1371/journal.pgph.0000075 (PMC10021618; doi:10.1371/journal.pgph.0000075)

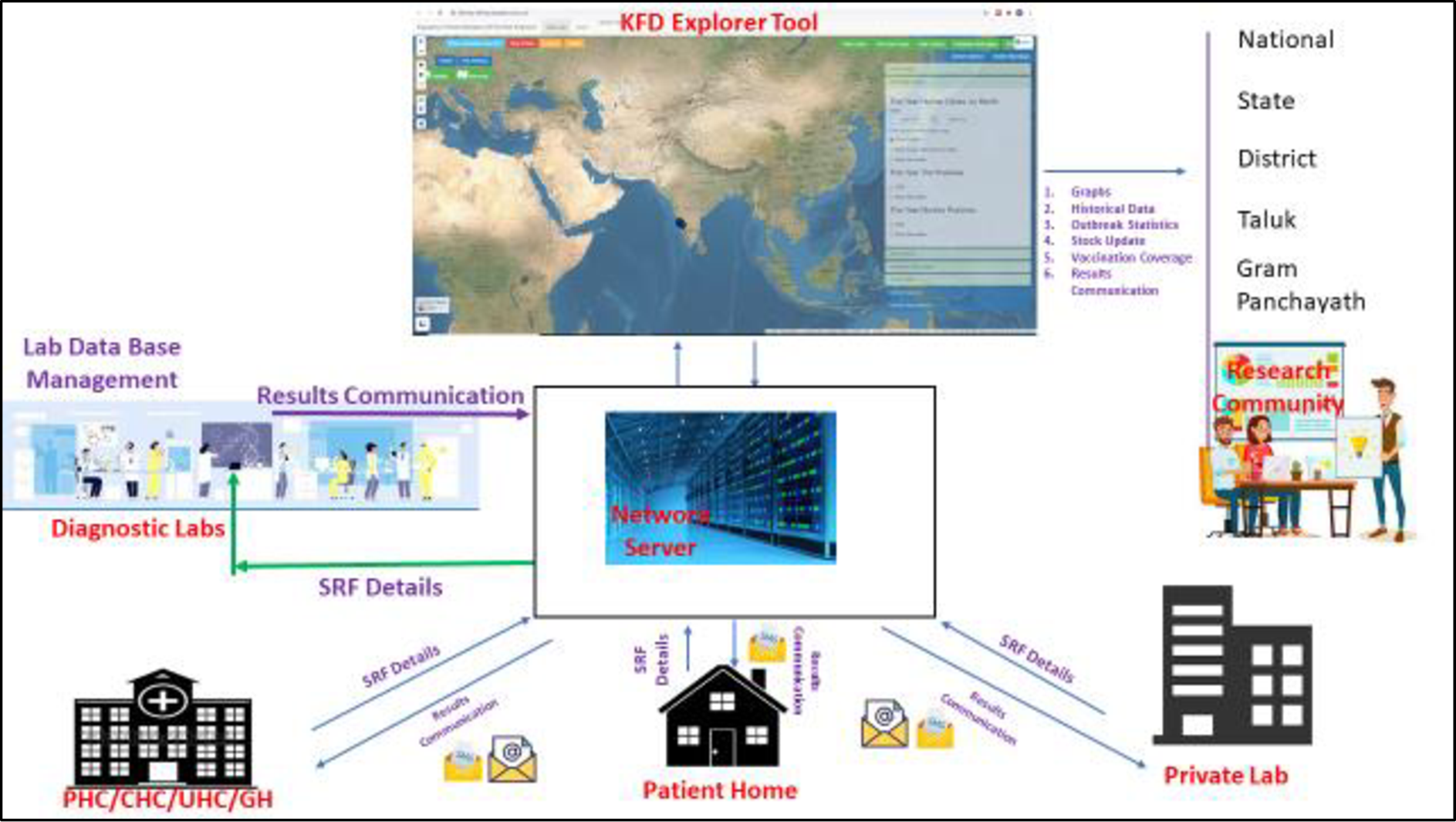

Supplement: S1 Fig — (TIF) [file pgph.0000075.s001.tif]
