# Supplementary material for: Co-production of knowledge as part of a OneHealth approach to better control zoonotic diseases
Source: PLOS Glob Public Health. 2022 Mar 24;2(3):e0000075. doi: 10.1371/journal.pgph.0000075 (PMC10021618; doi:10.1371/journal.pgph.0000075)
Supplement: S1 Table — (DOCX) [file pgph.0000075.s002.docx]

**Table S1. The characteristics of the workshop participants**

|  | **Participants** | **Sectoral Affiliation** | **State** | **District** | **Intervention level** |
| --- | --- | --- | --- | --- | --- |
| Workshop I | Participant 1 | Public Health (DHFWS) | Karnataka | Bangalore | State |
|  | Participant 2 | Public Health (DHFWS) | Karnataka | Shimoga | District |
|  | Participant 3 | Public Health (HFW) | Karnataka | Bangalore | State |
|  | Participant 4 | Animal Health (Veterinary College) | Karnataka | Shimoga | State |
|  | Participant 5 | Animal Health | Karnataka | Uttara Kannada | District |
|  | Participant 6 | Public Health | Maharashtra | Kolaphur | State |
|  | Participant 7 | Forest Department | Karnataka | Shimoga | District |
|  | Participant 8 | Public Health | Karnataka | Shimoga | District |
|  | Participant 9 | Family Welfare | Karnataka | Shimoga | District |
|  | Participant 10 | Animal Health | Karnataka | Shimoga | State |
|  | Participant 11 | Agriculture | Karnataka | Shimoga | District |
|  | Participant 12 | Animal Health | Karnataka | Shimoga | State |
|  | Participant 13 | Animal Health | Karnataka | Bangalore | State |
|  | Participant 14 | Animal Health | Karnataka | Bangalore | State ` |
|  | Participant 15 | Animal Health | Karnataka | Bangalore | State |
|  | Participant 16 | Animal Health | Karnataka | Bangalore | State |
|  | Participant 17 | Animal Health (DHFWS) | Karnataka | Shimoga | State |
|  | Participant 18 | Animal Health | Karnataka | Bangalore | State |
|  | Participant 19 | Public Health | Karnataka | Bangalore | State |
|  | Participant 20 | Animal Health | Kerala | Malapuram | District |
|  | Participant 21 | Public Health | Karnataka | Shimoga | District |
|  | Participant 22 | Public Health | Karnataka | Shimoga | District |
|  | Participant 23 | Public Health | Karnataka | Shimoga | District |
|  |  |  |  |  |  |
| Workshop II | Participant 1 | Animal Health | Karnataka | Bangalore | State |
|  | Participant 2 | Public Health | Karnataka | Shimoga | District |
|  | Participant 3 | Public Health | Karnataka | Shimoga | District |
|  | Participant 4 | Public Health | Karnataka | Shimoga | District |
|  | Participant 5 | Public Health | Karnataka | Kodugu | District |
|  | Participant 6 | Public Health | Karnataka | Shimoga | Taluk |
|  | Participant 7 | Public Health | Karnataka | Shimoga | Taluk |
|  | Participant 8 | Public Health | Karnataka | Shimoga | Taluk |
|  | Participant 9 | Animal Health | Karnataka | Shimoga | Taluk |
|  | Participant 10 | Animal Health | Karnataka | Bangalore | State |
|  | Participant 11 | Public Health | Karnataka | Bangalore | State |
|  | Participant 12 | Public Health | Karnataka | Bangalore | State |
|  | Participant 13 | Public Health | Goa | Goa | State |
|  | Participant 14 | Public Health | Maharashtra | Sindudurg | District |
|  | Participant 15 | Public Health | Karnataka | Udupi | District |
|  | Participant 16 | Public Health | Karnataka | Hassana | District |
|  | Participant 17 | Public Health | Karnataka | Uttara Karnataka | District |
|  | Participant 18 | Public Health | Delhi | Delhi | Central |
|  | Participant 19 | Public Health | Karnataka |  | National |
|  | Participant 20 | Public Health | Kerala | Thiruvananthapuram | State |
|  | Participant 21 | Forestry | Karnataka | Shimoga | District |
|  | Participant 22 | Forestry | Karnataka | Shimoga | District |
|  | Participant 23 | Forestry | Karnataka | Shimoga | District |
|  | Participant 24 | Forestry | Karnataka | Shimoga | District |
|  | Participant 25 | Public Health | Karnataka | Bangalore | District |
|  | Participant 26 | Forestry | Karnataka | Shimoga | District |
|  | Participant 27 | Public Health | Karnataka | Chamarajanagar | District |
|  | Participant 28 | Public Health | Kerala | Wayanad | District |
|  | Participant 29 | Forestry | Karnataka | Bangalore | State |
|  | Participant 30 | Public Health | Karnataka | Bangalore | State |
|  | Participant 31 | Public Health | Maharashtra | Kolhapur | District |
|  | Participant 32 | Public Health | Karnataka | Bangalore | State |
|  | Participant 33 | Public Health | Karnataka | Bangalore | State |
|  | Participant 34 | Public Health | Karnataka | Bangalore | State |
|  | Participant 35 | Public Health | Karnataka | Mysur | State |
