# Supplementary material for: Co-production of knowledge as part of a OneHealth approach to better control zoonotic diseases
Source: PLOS Glob Public Health. 2022 Mar 24;2(3):e0000075. doi: 10.1371/journal.pgph.0000075 (PMC10021618; doi:10.1371/journal.pgph.0000075)
Supplement: S2 Table — (DOCX) [file pgph.0000075.s003.docx]

**Table S2. Prioritisation of KFD risk factors identified by stakeholders and what changed or was strengthened over the co-production workshops**

| Ranking | Risk factors | Number of votes | How risks were addressed in project |
| --- | --- | --- | --- |
| 1 | Lack of education/awareness | 10 | Tick information cards were produced to inform local communities about risks from ticks and tick protection measures. Development of an educational video in progress. |
| 2 | Under or late reporting of monkey deaths | 9 | Accounted for in data interpretation in risk modelling |
| 2 | Deforestation and/or forest degradation | 9 | Integrated as a risk factor in models |
| 2 | Lack of awareness of preventative measures (tick repellants, vaccination) | 9 | Measured in cross-sectional household surveys WP2  Tick information cards produced (see above). |
| 3 | Lack of awareness or understanding of alternative hosts | 8 | Addressed in household and ecological surveys |
| 4 | Human use of forests | 7 | Addressed in household surveys and in spatial risk modelling |
| 4 | Low vaccination coverage | 7 | Addressed in household surveys and in spatial risk modelling |
| 4 | Poor diagnostics and surveillance | 7 | Improving surveillance and diagnostics is not a direct project aim but could result from a strengthened OneHealth network. Ecological analysis of vector and alternate hosts will inform surveillance strategies. |
| 4 | Lack of OneHealth policy | 7 | Project established a OneHealth WhatsApp network on KFD, project members attended National and State level technical committees on KFD and discussed OneHealth approach |
| 5 | Poor data management | 6 | The project provided a blueprint for future data management on KFD, for example ensuring that cases were georeferenced at a household level to capture landscape conditions favouring spillover |
| 5 | Poor understanding of tick ecology | 6 | Addressed in ecological surveys |
| 6 | Side effects and concerns about vaccines | 5 | Measured as part of the household surveys but not a direct research project aim |
| 7 | Living in or around forests | 4 | Addressed in risk modelling, household surveys and ecological surveys (stratified by forest proximity) |
| 7 | Favorable environment for ticks | 4 | Addressed in ecological surveys (habitat associations were measured) |
| 7 | Poor tick identification | 4 | Addressed in ecological research and capacity building (see Table 2) |
| 8 | Animal (livestock) grazing in forests | 2 | Addressed in household surveys and in risk factors integrated into risk modelling |
| 8 | Being in a vulnerable group: women, children, elderly, poor, health workers | 2 | Addressed in household surveys |
| 8 | Livestock movement (long range) | 2 | Not addressed directly in project, except in data interpretation in ecological research |
| 8 | Migratory birds | 2 | Not addressed directly in project, except in data interpretation in WP3 |
| 8 | Poor vaccine quality/efficacy | 2 | Not direct aim of project, though community perceptions of this were measured in the household surveys |
| 9 | Invasive vegetation | 1 | Addressed in ecological research (habitat associations) |
| 9 | Contact with rodents | 1 | Measured in ecological surveys (habitat associations) and household surveys |
| 9 | Exposure to monkeys | 1 | Measured in ecological surveys (habitat associations) and household surveys |
| 9 | Contact with ticks | 1 | Measured in ecological surveys (habitat associations) and household surveys |
| 9 | Lack of tick control | 1 | Measured in household surveys |
| 9 | High numbers of ticks | 1 | Measured in ecological surveys (habitat associations) |
| 10 | Flavivirus | 0 | Co-location of KFD with other zoonotic diseases addressed in risk modelling |
| 10 | Lack of health workers for KFD virus | 0 | Measured in household surveys and integrated as risk factors in risk models |
