# Supplementary material for: Co-production of knowledge as part of a OneHealth approach to better control zoonotic diseases
Source: PLOS Glob Public Health. 2022 Mar 24;2(3):e0000075. doi: 10.1371/journal.pgph.0000075 (PMC10021618; doi:10.1371/journal.pgph.0000075)
Supplement: S1 Text — (DOCX) [file pgph.0000075.s004.docx]

S1 Text. Self-completion questionnaire for Workshop participants

**Optimizing forest benefits whilst minimizing impacts of emerging zoonotic diseases: co-developing an interdisciplinary tool for forests in India**

**Self-completion questionnaire**

Thank you for taking time to complete this questionnaire. The purpose of this questionnaire is to obtain insights of cross-sectional actors (particularly from the Wildlife and Forestry Department, Agriculture and Horticulture Department, Education Department and the Tourism Department) about the potential management uses of the prototype Decision Support Tool and improvements required. The overarching aim of our research is to reduce health, welfare and livelihood impacts of Kyasanur Forest Disease by co-developing decision support tools and guidance based on improved scientific understanding of risk factors, with the wide-ranging actors and beneficiaries that interact with the disease system across public health, animal health and forestry sectors.

**General Information**

1. Could you tell us about your experience and role?

____________________________________________________________________________________________________________________________________________________________________________________________________________________________________________________________________________________________________________

1. Does your role include zoonotic disease surveillance or management? Yes/No
2. Are you involved in KFD management? Yes/No

**Current use of informatics for KFD management**

1. Do you utilise any decision support tools or risk maps in your work? Yes/ No
2. If YES is selected, please explain how you utilise the tools:

_________________________________________________________________________________________________________________________________________________________________________________________________________________________________

If NO is selected, please explain why:

_________________________________________________________________________________________________________________________________________________________________________________________________________________________________

1. What would you say are the advantages of the Shiny App DST over other existing tools? [*To be asked after respondents have had the opportunity to familiarise themselves with the tool*]

_______________________________________________________________________________________________________________________________________________________________________________________________________________

1. How likely are you to use the Shiny App DST to assist in your work?

1 – Very likely 2 – Likely 3 – Unsure 4 – Unlikely 5 – Not at all likely

1. Please may you explain your response?

____________________________________________________________________________________________________________________________________________________________________________________________________________________________________________________________________________________________________________

1. In your opinion, which stakeholder groups are likely to use the App?

____________________________________________________________________________________________________________________________________________________________________________________________________________________________________________________________________________________

1. In your view, how can widespread awareness about the App be achieved?

____________________________________________________________________________________________________________________________________________________________________________________________________________________________________________________________________________________

**Functionality characteristics of the Shiny App DST**

1. In its current presentation, how would you describe the user-friendliness of the Shiny App DST

Very user-friendly [ ] User-friendly [ ] fairly user-friendly [ ] Not user-friendly [ ]

1. Please may you explain your answer:

_________________________________________________________________________________________________________________________________________________________________________________________________________________________________

1. In its current presentation, how would you describe the content of the Shiny App DST

Very informative [ ] Informative [ ] Quite informative [ ] Not at all informative [ ]

1. In its current presentation, which parts or features of the App:
2. are additional to any decision process or risk assessment you currently use?

_______________________________________________________________________________________________________________________________________________________________________________________________________________

1. are most useful or informative for your work? Why do you say that?

___________________________________________________________________________________________________________________________________________________________________________________________________________________________________________________________________________________________________________________________

1. would you find difficult to assess and why?

___________________________________________________________________________________________________________________________________________________________________________________________________________________________________________________________________________________________________________________________

1. Are there any additional functionalities or data you think may be useful to improve the usefulness that we should explore? If so, please explain:

_________________________________________________________________________________________________________________________________________________________________________________________________________________________________________________________________________________________________________________________________________________________

1. What do you see as the benefits of adding such functionality or data to the tool, if any, to your management?

____________________________________________________________________________________________________________________________________________________________________________________________________________________________________________________________________________________

**Key health system/ ecological/environmental/social aspects to be added to the tool.**

1. In your opinion, are there additional information that should be added to the tool to enhance its usefulness? If so, please indicate the suggested additional information:

____________________________________________________________________________________________________________________________________________________________________________________________________________________________________________________________________________________________________________

**Potential Data Issues**

1. In your opinion, who should be granted access to the tool?

____________________________________________________________________________________________________________________________________________________________________________________________________________________________________________________________________________________

1. In your opinion, how data should be synchronised with the IDSP/ IHIP?

____________________________________________________________________________________________________________________________________________________________________________________________________________________________________________________________________________________

1. Did you attend any of our prior MonkeyFeverRisk workshops? Yes/No
2. If Yes is selected, do you feel your feedback has been integrated in the new version of the Shiny App DST?

_______________________________________________________________________________________________________________________________________________________________________________________________________________

**Personal Information**

1. Name of respondent____________________________________________________
2. Designation/Job title ___________________________________________________
3. Qualification__________________________________________________________
4. Specialisation_________________________________________________________
5. Organisation/ Sectoral affiliation_________________________________________________
6. Geographical area/ scale of operation: State [ ] District [ ] Other_____________________
7. Telephone Number ____________________________________________________
8. Email Address_________________________________________________________

Many thanks for your participation in our research.
