# Supplementary material for: Co-production of knowledge as part of a OneHealth approach to better control zoonotic diseases
Source: PLOS Glob Public Health. 2022 Mar 24;2(3):e0000075. doi: 10.1371/journal.pgph.0000075 (PMC10021618; doi:10.1371/journal.pgph.0000075)
Supplement: Attachment — Submitted filename: Response to Reviewers Comments_Final.docx [file pgph.0000075.s005.docx]

**Response to Reviewer Comments – ‘Co-production of knowledge as part of a OneHealth approach to better control zoonotic diseases’ – PLOS Global Public Health – PGPH-D-21-00480**

**Overview of changes made**

We would like to thank the editor and reviewers for the careful and thorough reading of our manuscript and for the thoughtful comments and constructive suggestions, which help improve the quality of the manuscript. We have made substantive changes to the paper in response to these reviews.

**Additional editor Comments:**

1. At the outset, I take this opportunity to thank you personally, and all the reviewers recognize your hard work and motivation to charter in publishing the interdisciplinary work. Two of the reviewers have expressed that this manuscript merits an opportunity to publish provided the report of the results is modified and the manuscript is revised. One of the reviewers has pointed to self-citations and adapting another figure from your published manuscript. Please make the modifications as suggested by the reviewer.

*Response: Thank-you. We really appreciate the positive comments particularly highlighting the value of the paper in advancing interdisciplinary work within the context of One Health. We are also grateful for the very thoughtful suggestions, which we addressed as detailed below:*

2. At the conceptual level, we recognize that your team has profound knowledge of One Health. The rationale for the co-production process conducted against the established systems approach in One Health needs to be explained. I will be happy to receive a revised manuscript addressing the specific recommendations by the reviewers.

*Response: We again appreciate the editor’s acknowledgement of our team’s complementary expertise and knowledge on OneHealth. We agree that a systems approach undergirds OneHealth and that our rationale for implementing a co-production approach requires further clarification. At the outset and throughout the execution of our interdisciplinary OneHealth Indo-UK partnership, the MonkeyFeverRisk project (*[*https://www.monkeyfeverrisk.ceh.ac.uk/*](https://www.monkeyfeverrisk.ceh.ac.uk/)*), from which the present study is derived, a systems approach within a OneHealth paradigm was applied in the overall conceptualisation, research team constitution and implementation. In this context, our interdisciplinary project team worked together with disease managers and policy-makers across the public health, animal health, and environment sectors to improve our understanding of the multiple risk factors of Kyasanur Forest Disease (KFD) and co-produce guidance and decision-support tools for KFD management. A OneHealth system approach (alluding to the individual roles of sectoral actors in understanding the KFD system) thus undergirded our overall project design and implementation, informing our engagement with cross-sectoral stakeholders. As explained in the introduction (page 5, lines 114-118), our co-production approach embedded within the overall OneHealth systems thinking essentially highlight the strengths of the different stages of the framing, implementation and development of tools. The adoption of a co-production approach is not necessarily a departure from a OneHealth systems thinking, but represents an iterative and collaborative process involving transdisciplinary expertise, knowledge and cross-sectoral actors to produce context-specific knowledge and tools to improve KFD surveillance and management. In essence, our implementation of a co-production approach was embedded within a broader systems approach which afforded meaningful engagement of cross-sectoral stakeholders in all framing, implementation and development phases of decision-support tool and risk maps from the project.*

**Reviewers’ comments to the Author**

**Reviewer #1:**

**General comments**

The co-production initiative that includes several stakeholders from public health, animal health and environment to create a DST would be very beneficial for the control of zoonotic diseases such as KFD. The information generated from the manuscript is highly relevant for future decision makers in One Heath. The authors did a great job in explaining the co-production process and how they could contribute to the future disease outbreak management.

*Response: Thank you for the positive review and very thoughtful suggestions, which we addressed as detailed below:*

However, the manuscript lack specific details on how the data were analyzed that resulted in the development of DST. Here are few suggestions for the authors

1. How were the stakeholders identified? Where they involved in policy/decision making process. It would be better to list out the title of the stake holders who attended both the workshops.

*Response: On page 10 (lines 212-232) of the revised manuscript, we have added a new paragraph that elucidates the process of stakeholder identification and selection of workshop participants. Please see the related sentences below:*

*“Prior to organising the workshops, the research team conducted a stakeholder mapping exercise, which culminated in a list of current and potential cross-sectoral actors involved in KFD management at local, national or state level. Based on this stakeholder list, workshop participants were purposively selected to warrant (a balanced) representation of policy/decision-makers from the human health, animal health and forestry sectors with designated roles in KFD management, who had the potential to provide relevant, detailed and diverse information about the KFD system. The variety of sectoral representation, participants’ experience with KFD prevention and control, and the ability to assess the value of the DST and other project outputs were the main criteria for inclusion of participants.*

*In recruiting prospective participants for the workshops, formal introductory letters were sent to the appropriate state departmental directorates (in accordance with institutional protocols), explaining the objectives of the overall project, and requesting them to depute up to 7 staff from their respective departments to attend the workshops. The introduction letters were sent to the Karnataka state directorate of health and the NCDC (public health sector), Department of Animal Husbandry and Veterinary Services (DAHD) (animal health sector) and State Forest Department (environment sector) respectively. Two of the co-authors (MMC and SLH) followed up with telephone calls to further explain the purpose of the workshops and confirm participation at the sessions, in accordance with administrative protocol and local custom. The characteristics of the workshop participants are outlined in Table S1 (see supplementary material).”*

2. The authors should describe the approach used to develop desk-based spatial DST mentioned in line 219

*Response: Thank you for the comment. In between the framing workshop and the knowledge and experimentation workshop, the desk-based spatial DST was developed using a multi-pronged approach. Firstly human case data were collated across the KFD-affected areas and modelled in relation to risk factors identified by stakeholders or in the scientific literature to produce and validate risk maps for disease spill-over (as described in detail in Purse et al. 2020). The desk-based decision support tool (“KFDExplorer tool”) was then constructed, integrating surveillance and landscape data, alongside the risk maps, accounting for the stakeholder preferences identified in the framing workshop for how seasonal and geographical information should be presented and overlaid to inform management. Importantly, a post-doctoral researcher from the team was embedded in the health department throughout this process and alongside data collation and modelling was involved in the KFD response, participating in interventions such as inter-departmental sensitisation workshops for decision makers across sectors, and community awareness raising activities. This allowed for further reciprocal knowledge integration and feedback on utility and contextualisation of the developing project DST and risk guidance in between the project workshops.*

3. The authors also need to give a detailed explanation on how the data generated from the two workshops were analyzed. For example how was the risk factors identified and how did they account of the unequal participation from different sectors (6 participants from public health vs 10 participants from animal health in Workshop 1)

*Response: Thank you for the comment, we accept that further explanation on how the collated data from the workshops were analysed. On pages 14-15 (lines 331-342), we have added a paragraph highlighting that the procedure for workshop data generation and analysis. Please see the added sentence below:*

*“The collated qualitative data from the workshops (including audio transcriptions, observation notes and suggestions made by participants) were analysed using thematic and content analysis approach. After transcribing the audio recordings of the workshop discussions, the transcripts were manually coded (line by line and axial) independently by three of the co-authors (JCY, MAO and FAA), following Braun & Clark’s (2006) thematic content analysis approach. Exemplar quotations from participants and observations of the facilitating team were also collated. Each reviewer (JCY, MAO, FAA) generated themes and sub-themes, which were subsequently compared and refined to produce the final thematic analysis. If there were any disagreements, reviewers discussed themes to collectively agree on revisions. All participant information were de-identified using pseudonyms for the analysis and report-writing; these pseudonyms are reported here.”*

4. What was the criteria used by the authors to either include or exclude the risk factors (line 278) and how was the users and uses identified by the stakeholders screened to select for the key potential users and uses (Table 3).

*Response: Thank you for the comment. Purse et al. (2020) referenced in the revised manuscript describes in full which of the risk factors identified by stakeholders were included or excluded. As mentioned in the revised manuscript (see pages 11-12, lines 253-261), risk factors were included if spatial datasets or proxies existed for them and excluded otherwise. Robust statistical procedures were used to test the relative importance of the included risk factors in determining spill-over and independent validation data (from years following those used to train the model) were used to determine the predictive accuracy of the resulting maps of spill-over.*

5. Is there any link for accessing the DST tool so that the reader could have a better understanding about the usefulness of the approach

*Response: Thank you for the comment and particular interest in the DST tool. Given that the DST contains household level data for human cases and in compliance with ethical stipulations and Indian Council for Medical Research (ICMR) guidelines, the DST is not intended to be publicly accessible but will only be available to those who will be involved in KFD management. We, however, are contemplating running training workshop (on an abridged version of the DST which is yet to be developed) to interested researchers as part of our ongoing dissemination of our project outputs.*

**Reviewer #2:**

**General comments**

It is a well written manuscript. There are a few punctuation errors that require attention. The introduction could be made more crisp and to the point. In the manuscript as a whole, there are a few reiterations of things that have already been touched upon. The manuscript will benefit from an editing to make the entire thing seem more put-together and without any repetitions.
Kindly do the needful.

*Response: Thank you for the positive review of our manuscript and thoughtful suggestions. We have edited the entire manuscript, shortened the introduction and corrected punctuation errors as suggested by the reviewer.*

**Reviewer #3:**

**Title**

If this co-production of knowledge is from a case study of southern India, then this should reflect on the title.

*Response: Thank you for the comment. We however feel that the paper (once published) will be of interest to a wider OneHealth audience and as such having a broad title affords its potential visibility across different scientific databases. In any event, we have also captured India and Kyasanur Forest Disease as part of the keywords which we feel is sufficient for any targeted/ situated bibliographic searches.*

**Abstract**

1. L29: I could rather say, necessarily not working together. Although there might be some knowledge, and they may work under certain circumstances, this is not necessarily happening.

*Response: Thank you for the comment. However, we feel that the original phrasing “not usually work together” is better in this context as changing to “necessarily not” may be misconstrued as “of necessity not”.*

2. L32: reduction of disease impact or risk mitigation at the interface of human-animal-environment. The author may like to justify why they are saying the reduction of disease impact rather than risks.

*Response: On page 2 (line 32) of the revised manuscript, we have added the phrase “and/or mitigate risks” as suggested by the reviewer. In this context, we are discussing impact because the effectiveness of interventions may determine disease impact rather than altering risk of spill-over. Indeed, we reported in recent project-related study on KFD that access to disease information improved households’ propensity to implement adaptation strategies, which ultimately reduced disease impact in affected community (see Asaaga et al. 2021, PLOS NTDs -* [*https://doi.org/10.1371/journal.pntd.0009265*](https://doi.org/10.1371/journal.pntd.0009265)*)*

3. L37: If the case study is about KFD, that must align with the L32 statement and reflect in the title.

*Response: Thank you for the comment. We however respectfully disagree with the reviewer as we feel that by specifying Kyasanur Forest Disease (KFD) in the abstract it will still be picked up in search engines. Besides, the present study is of wider interest beyond KFD.*

**Introduction**

1. L55-56: It seems the sentence is not well placed.

*Response: On page 3 (lines 56-57) of the revised manuscript, we have revised the sentence in accordance with the reviewer’s suggestion. The revised sentence now reads:*

*“Globally, a majority of poor communities depend on healthy ecosystems for livelihoods, welfare and food security particularly in LMICs.”*

2. L65-68: Difficult to generalize, as it strongly depends on the governance structure and the functionalities. Yes, it's true if it has been written from the Indian perspective, but authors need to specify here.

*Response: We agree that the extent of cross-sectoral coordination for zoonoses control is context dependent.	There is also empirical evidence supporting the view that rapid action with respect to zoonoses control is often deterred by poor evidence-bases and limited coordination between relevant stakeholders in LMICs, including India (see Asaaga et al. 2021b:* [*https://doi.org/10.1186/s12889-021-11545-7*](https://doi.org/10.1186/s12889-021-11545-7)*, Chatterjee et al. 2016:* [*https://doi.org/10.1186/s40249-016-0181-2*](https://doi.org/10.1186/s40249-016-0181-2)*). On page 3 (lines 64-67), we have rephrased the sentence to help clarify this observation. The revised paragraph now reads:*

*“Despite increased global and national attention on the need for effective strategies for zoonoses control , rapid action is often deterred by poor evidence-bases and limited coordination between stakeholders who do not normally act in concert (e.g. animal health, public health, forest departments), particularly in LMICs including India (Paul et al. 2020; Asaaga et al. 2021b).*

3. L74: Why ‘global’. One Health is local too. So why global One Health?

*Response: Thanks for the comment. We agree that OneHealth is both global and localised too. Indeed, we are aware of recent studies reporting on OneHealth operationalisation in a city context in India (please see: Yasobant et al. 2018* [*https://doi.org/10.1186/s12961-018-0398-6*](https://doi.org/10.1186/s12961-018-0398-6)*; Yasobant et al. 2019* [*https://doi.org/10.1371/journal.pone.0220152*](https://doi.org/10.1371/journal.pone.0220152)*; Yasobant et al. 2021* [*https://doi.org/10.1186/s12960-021-00558-3*](https://doi.org/10.1186/s12960-021-00558-3)*). The usage of “global” in this context is thus intended to depict the widespread application of OneHealth across different contexts and not necessarily to suggest it cannot be operationalised at a local scale.*

4. L77: The One Health scope is not just to reduce the disease impact. It must be valued as an integrated risk management perspective too.

*Response: We agree that the OneHealth scope transcends reduction of disease impact. We have rephrased line 80 to acknowledge the added value from an integrated risk management perspective as suggested by the reviewer (see page 4, line 80). The revised sentence now reads:*

*“The global OneHealth paradigm is an approach which recognises the “interconnectedness of human health, wildlife and domestic animal health and the environment” and the value of evaluating these interactions in an integrated, cross-sectoral and transdisciplinary manner to reduce pathogen transmission risk and mitigate disease impacts (Asokan & Asokan, 2016; Kelly et al., 2017; Lebov et al., 2017).”*

5. L80-83: Which models authors are talking about? Need clarification

*Response: Apologies for the confusion. On page 4 (line 84) of the revised manuscript, we have clarified that the models in this context refers to risk models.*

6. Overall well-written background; however, I have a gross critique. Why are authors introducing the concept of co-production here? In the rationale of the One Health approach, cross-sectoral collaboration is the base, and the theme lies with the systems approach. There must be a strong argument, how this co-production process is different from the systems approach of solving multi-dimensional issues, which is needed in the background.

*Response: Thank you for the comment. Please see our response to editor’s additional comment point 2.*

*We agree that a systems approach undergirds the OneHealth and that our rationale for implementing a co-production require further justification. We have principally addressed this comment by adding a paragraph to the introduction (see page 5, lines 114 -118) on the rationale for co-production process, highlighting the former is not a substitute for a systems approach per se but actually embedded later. We further argue that a co-production process within One Health context offers a practical lens for iterative and collaborative engagement of cross-sectoral actors (e.g. human health, animal health and forestry) to co-create knowledge and tools. In so doing, the co-production process help to highlight the strength of the different stages of the framing, implementation and development of tools from a systems perspective to holistically understand roles and responsibilities of respective actors within the KFD system.*

**Methods**

1. L156-187: I could see multiple self-citations (please refer to other literature).

*Response: Thank you for the comment. Although we acknowledge instances of self-citation, we do not see this as a problem or lack of referencing other related literature. Indeed, we submit that the few instances where we are self-citing is particularly to help readership appreciate the context of existing work that the present study builds on and not to merely self-cite per se. On page 16 (line 368) of the revised manuscript, for example, we have added phrases like for detailed description of the modelling approach adopted, see Purse et al. 2020* [*https://doi.org/10.1371/journal.pntd.0008179*](https://doi.org/10.1371/journal.pntd.0008179) *to help clarify our position and rationale for self-citing.*

2. Rather than writing about the whole disease management system here in the method section. It will be great to replace the KFD disease management system with a three-tier approach of central-state-local governance and all the stakeholders involved in this process.

*Response: Thank you for the comment. We however respectfully disagree with the reviewer. A central element of our approach was to map and engage with system specific actors and generate a tool that is of use to their decision making and aligns with their priorities. We are not yet ready (in the position) to extend this to multiple disease burdens, for which the more generalised actor network would be needed. More over the focus on central-state-local governance may miss important actors. Indeed, our team has recently published a related manuscript on operationalisation of OneHealth in India that adopts such central-state –local governance characterisation (please see: Asaaga et al. 2021b:* [*https://bmcpublichealth.biomedcentral.com/articles/10.1186/s12889-021-11545-7*](https://bmcpublichealth.biomedcentral.com/articles/10.1186/s12889-021-11545-7)*)*

3. Fig.1: It’s the same figure from author’s recently published article. Can I suggest developing a new framework specific to KFD, although the aim is to provide a generic system to readers?

*Response: We agree that the figure 1 is adapted from a recent published article by our team, which we duly acknowledged in the original manuscript. The rationale for this was to help give a pictorial impression of the complexity of the KFD system and actors involved. We have however, developed a new framework for KFD in line with the reviewer’s suggestion (see page 9 of the revised manuscript). Please see the new framework below:*

*Fig.1 A simplified illustration of the sectors and the politico-administrative actors of the KFD management in India including actors from each sector that are impinging on the system or are impacted by KFD in each sector.*

4. L210-248: It is important how researchers invited these participants. In the participatory approach, it is an integral component as the outcome depends on who participated and the rank of the stakeholders, including experiences. Please explain and justify why there is a discrepancy in the number of stakeholders from three domains of One Health. Also, add a para on the pre-workshop-related development works.

*Response: Thank you for the comment. On page10 (lines 212-232) we have added a paragraph clarifying the participant selection process. Suffice to say that stakeholders were invited by formally requesting relevant departmental heads to depute, in accordance with the administrative protocol and custom. Although we planned to have a balanced representation across sectors, this was not achieved due circumstances beyond the control of the project team. For instance, the participation at the first workshop was influenced by the flooding event which occurred in parts of Karnataka and Kerala leading to the withdrawal of some invitees who had hitherto confirmed their participation. Moreover, the second workshop had a disproportionate representation of Public Health actors principally due to a specific request to participate received from health department officials stationed at Sagara (a KFD endemic area). The project team granted this request as it was deemed a good window of opportunity to obtain feedback on the prototype desk-based spatial DST as well as to build good rapport for on-ward dissemination and uptake of project outputs (see lines 285-300, page 13 of revised manuscript). Please see the added paragraph below:*

*“Despite efforts to achieve a balanced sectoral representation at both workshops, this was not achieved due to circumstances beyond the control of the project team. A case in point is the major flooding event which occurred in parts of Kerala and Karnataka states (coinciding with the first workshop), which resulted in the withdrawal of some invitees who had hitherto confirmed their participation. Besides, the first workshop clashed with a field programme of the forest department, which meant that some initially deputed officials had to decline the invitation to attend the workshop. The second workshop had a disproportionate representation of Public Health actors principally due to the specific expression of interest to participate received from the health departments officials stationed at Sagara (an area newly affected by KFD that season). The project team granted this request as it was deemed a good window of opportunity to obtain feedback on the prototype desk-based spatial DST as well as build good rapport for on-ward dissemination and uptake of project outputs. Nonetheless, further follow-up interviews and conversations with under-represented stakeholders (principally from the forestry department) during the course of the project (including the national KFD stakeholder consultation workshop) afforded in-depth understanding of their perspectives and inputs into the DST development.”*

**Results**

1. L271-284: Is there any decision-making tree analysis or hierarchical analysis conducted as part of the problem prioritization? If so, please specify. If not, then justify the process. And how the weightage calculation (if any) is conducted needs to be expanded. If not, then why there was no such process done, justify.

*Response: On page 11 (lines 234-252) of the revised manuscript, we have added a new paragraph that further elucidates the problem prioritisation process during the stakeholder workshops. In brief, we employed the nominal group technique (NGT), a participatory consensus-building approach (during the multi-stakeholder workshops) to help generate and prioritise ideas and ensure equal participation of the diverse stakeholder groups. In essence, the NGT involved a facilitated discussion and simple voting process (i.e. rank-ordering of risk factors and priorities) which helped create a list of collectively established priorities for the spatial DST development. Aside from the process helping circumvent the typical power dynamics of diverse stakeholder groups, it also ensured quick gathering of sizable amount of data within a short time frame in a single event as well as alleviated possible researchers’ biases as data was interpreted by participants.*

2. L303-341: Readers may like to have a brief idea of the KFD project outline prior to the workshop. And how that priority has changed over the co-production workshops.

*Response: Thank you for the comment. We have added a table as part of supplementary information (Table S2) outlining a complete list of ranked risk factors as identified by stakeholders and what changed and/or strengthened over the course of the multi-stakeholder workshops as suggested by the reviewer.*

**Table S2. Prioritisation of KFD risk factors identified by stakeholders and what changed or was strengthened over the co-production workshops**

| Ranking | Risk factors | Number of votes | How risks were addressed in project |
| --- | --- | --- | --- |
| 1 | Lack of education/awareness | 10 | Tick information cards were produced to inform local communities about risks from ticks and tick protection measures. Development of an educational video in progress. |
| 2 | Under or late reporting of monkey deaths | 9 | Accounted for in data interpretation in risk modelling |
| 2 | Deforestation and/or forest degradation | 9 | Integrated as a risk factor in models |
| 2 | Lack of awareness of preventative measures (tick repellants, vaccination) | 9 | Measured in cross-sectional household surveys WP2  Tick information cards produced (see above). |
| 3 | Lack of awareness or understanding of alternative hosts | 8 | Addressed in household and ecological surveys |
| 4 | Human use of forests | 7 | Addressed in household surveys and in spatial risk modelling |
| 4 | Low vaccination coverage | 7 | Addressed in household surveys and in spatial risk modelling |
| 4 | Poor diagnostics and surveillance | 7 | Improving surveillance and diagnostics is not a direct project aim but could result from a strengthened OneHealth network. Ecological analysis of vector and alternate hosts will inform surveillance strategies. |
| 4 | Lack of OneHealth policy | 7 | Project established a OneHealth WhatsApp network on KFD, project members attended National and State level technical committees on KFD and discussed OneHealth approach |
| 5 | Poor data management | 6 | The project provided a blueprint for future data management on KFD, for example ensuring that cases were georeferenced at a household level to capture landscape conditions favouring spillover |
| 5 | Poor understanding of tick ecology | 6 | Addressed in ecological surveys |
| 6 | Side effects and concerns about vaccines | 5 | Measured as part of the household surveys but not a direct research project aim |
| 7 | Living in or around forests | 4 | Addressed in risk modelling, household surveys and ecological surveys (stratified by forest proximity) |
| 7 | Favorable environment for ticks | 4 | Addressed in ecological surveys (habitat associations were measured) |
| 7 | Poor tick identification | 4 | Addressed in ecological research and capacity building (see Table 2) |
| 8 | Animal (livestock) grazing in forests | 2 | Addressed in household surveys and in risk factors integrated into risk modelling |
| 8 | Being in a vulnerable group: women, children, elderly, poor, health workers | 2 | Addressed in household surveys |
| 8 | Livestock movement (long range) | 2 | Not addressed directly in project, except in data interpretation in ecological research |
| 8 | Migratory birds | 2 | Not addressed directly in project, except in data interpretation in WP3 |
| 8 | Poor vaccine quality/efficacy | 2 | Not direct aim of project, though community perceptions of this were measured in the household surveys |
| 9 | Invasive vegetation | 1 | Addressed in ecological research (habitat associations) |
| 9 | Contact with rodents | 1 | Measured in ecological surveys (habitat associations) and household surveys |
| 9 | Exposure to monkeys | 1 | Measured in ecological surveys (habitat associations) and household surveys |
| 9 | Contact with ticks | 1 | Measured in ecological surveys (habitat associations) and household surveys |
| 9 | Lack of tick control | 1 | Measured in household surveys |
| 9 | High numbers of ticks | 1 | Measured in ecological surveys (habitat associations) |
| 10 | Flavivirus | 0 | Co-location of KFD with other zoonotic diseases addressed in risk modelling |
| 10 | Lack of health workers for KFD virus | 0 | Measured in household surveys and integrated as risk factors in risk models |

3. L388-424: Some of the recommendations are aligned with the findings of this workshop. The authors may like to shift those recommendations (perhaps overall and specific) towards the end of the draft with a separate section.

*Response: Thank you for the suggestion. In restructuring the manuscript (as suggested by Reviewer#5), we have reported the benefits and challenges from each stage of the co-production process (including related stakeholder opinions/suggestions) under the results section to ensure the lucidity of the overall argument. We reflect on the implications of these insights and lessons (beyond the KFD system) for implementing future co-production of zoonotic disease management in a OneHealth context.*

**Conclusions**

Authors may like to provide a guidance framework in the form of process flow, including the impact matrix for better understanding. Also, link factors that could potentially affect each step of the process flow and minimize those.

*Response: Thank you for the suggestion. We however feel that our retrospective theory of change framework on the researcher expectations and the system-level outcomes of the co-production process in conjunction with the detailed reflections on the lessons learned affords an in-depth understanding for future co-production of zoonotic disease tools and management.*

**Reviewer #4:**

**General comments**

This is an excellent article that makes a very timely contribution to our understanding of how to move the One Health approach from theory to practice. The structure of the paper is set out effectively and the overall argument supported well.

*Response: Thank you. We particularly appreciate the positive comments highlighting the value of our paper in advancing the operationalisation of the One Health approach. We are also grateful for the very thoughtful suggestions, which we addressed as detailed below:*

**Suggestions for revisions:**

1. In the methods section, there should be more information on how the data generated as part of the workshops was analyzed. Was it imported into Nvivo, or a similar qualitative research software? What type of analysis (thematic, constant comparative, discourse) was conducted? How were coding categories developed (inductively, deductively), etc?

*Response: Thank you for the comment. We agree that further information about the data gathering and analysis needed to help readership better understand our methodological approach. On pages 14-15 (lines 331-342) of the revised manuscript, we have added new section clarifying that the data generated were analysed manually following Braun and Clark’s (2006) six steps to thematic and content analysis methodology. Please see below the added section:*

*“2.3 Data analysis*

*The collated qualitative data from the workshops (including audio transcriptions, observation notes and suggestions made by participants) were* *thematically analysed within a deductive framework. After transcribing the audio recordings of the workshop discussions, the transcripts were manually coded (line by line and axial) independently by three of the co-authors (JCY, MAO and FAA), following Braun & Clark’s (2006) thematic content analysis approach. Exemplar quotations from participants and observations of the facilitation team were also collated. Each reviewer (JCY, MAO and FAA) generated themes and sub-themes, which were subsequently compared and refined to produce the final thematic analysis. If there were any disagreements, reviewers discussed themes to collectively agree on revisions. All participant information were de-identified using pseudonyms for the analysis and report-writing; these pseudonyms are reported here.”*

2. It is interesting that stakeholders wanted to have the DST be integrated with WHO's IHIP but that this was not done. Could you provide more details on why.

*Response: Thank you for the comment, we agree that the non-integration of the DST with WHO’s IHIP require clarification. On page 27, we have added a footnote#4 to help clarify that the project indeed contemplated the beneficence of such integration (and had initial consultations with the IHIP team), the current COVID-19 pandemic significantly delayed further progress in that regard. Please see the explanatory footnote#4 below:*

*“The strategy was to roll out MonkeyFeverRisk DST first independently of IHIP on Indian Government servers and then make arrangements with the WHO IHIP team for further integration, taking account of feedback from a broader range of end users during the KFD season. As at the time of submission of this paper, the first step has been delayed by the COVID-19 pandemic and the need for the e-hospital to manage COVID-19 data and provide tools to inform the response.”*

**Additional comments:**

1. P.9 Fig.1 Capture should be shortened and acronyms placed at end of article.

*Response: Thank you for the comment. We have shortened the caption of Figure 1 as suggested by the reviewer.*

2. P.22 line 329 Punctuation missing after the word direction

*Response: We have added a full stop after the word direction as indicated by the reviewer.*

3. P.27 Fig.3 Capture should be shortened and figure explained in text/footnote not heading

*Response: Thank you for the comment. We have shortened the title of figure 3 and added an explanatory footnote#3 (see page 25) as suggested by the reviewer.*

*“Fig.3 – the areas predicted to be highly suitable for spill-over of KFD to humans are highlighted in red versus areas predicted to be unsuitable for spill-over in blue. The right hand menus in green indicate how data on environmental risk factors, on KFDV-positive dead monkeys and KFDV-positive ticks, can be visualised alongside human case locations, whilst the use of a detailed base map depicts landscape contextual features that guide management such as villages and roads.”*

**Reviewer #5:**

**General Comments**

The manuscript presents an interesting case study of a scientific project attempting to engage in a more inclusive process with stakeholders of their work. The reflective work done to prepare this manuscript is highly valuable and should be acknowledged. It is a known struggle for scientists accustomed to quantitative research to present qualitative work with the same rigor. The progress in inter- and transdisciplinary health sciences will require much more of these pioneering efforts. I therefore commend this work for publication after some thorough revision.

*Response: Thank you. We really appreciate the positive comments particularly highlighting the value of the paper in advancing interdisciplinary work within the context of One Health. We are also grateful for the very thoughtful suggestions, which we addressed as detailed below:*

The article would benefit from some reorganisation according to the “standards for reporting qualitative research”. In particular, the result section should provide the data that the authors employ to draw their conclusions in the discussion section. As it stands, the article appears rather as an opinion piece than a scientific report from qualitative research. Also, the discussion should make substantially more reference to other work conducted in the field of one health and transdisciplinarity. Maybe, the authors could formulate a working hypothesis for the introduction of this work, which then would facilitate structuring the discussion.

*Response: Thank you for the comment. As suggested by the reviewer, we have re-organised the entire manuscript including the results and discussion sections.*

**Specific Comments:**

1. L59: The link between ecosystem degradation and disease burden needs some broader references looking into the problem, e.g. highly relevant references would be
   <https://www.frontiersin.org/articles/10.3389/fvets.2021.661063/full>; <https://doi.org/10.1016/j.biocon.2020.108707>

*Response: Thank you for the comment. We, however, feel that the suggested references may not be appropriate in this context as they are very broad scale correlational studies of links between deforestation and disease outbreaks, which though valid does not capture the mechanisms underpinning links and providing outputs that inform decision-making. Nonetheless, we have added two additional references –Plowright et al. 2021:* [*https://doi.org/10.1016/S2542-5196(21)00031-0*](https://doi.org/10.1016/S2542-5196(21)00031-0)*; Reaser et al. 2021:* [*https://doi.org/10.1111/rec.13357*](https://doi.org/10.1111/rec.13357) *(see page 3, lines 62-63 of the revised manuscript).*

2. L74-7: consider inverting the statement “...to endorse and facilitate health” instead of “…reduce the impact of diseases”. There are implications when focusing on diseases rather than health, particularly in regards to the stakeholders that are considered. When intending a one health approach, the objective should be positive rather than the absence of negative. For detailed rationale behind this, please see: <https://link.springer.com/article/10.1007%2Fs11625-019-00674-z>

*Response:* *Thank you for the suggestion. On page 4 (line 80) we have added the phrase “reduce pathogen transmission risk and mitigate disease impacts” to help clarify our argument.*

3. L86-9: In the context of One Health, the authors could consider also referring to transdisciplinarity as a term used to describe knowledge co-production, e.g. <http://www.ncbi.nlm.nih.gov/pubmed/20391395>;<http://link.springer.com/10.1007/s13280-012-0372-4>;<https://www.cabi.org/bookshop/book/9781789242577/>

*Response: As suggested by the reviewer, we have duly acknowledged transdisciplinarity as an alternative terminology used to describe knowledge co-production (see page 4, footnote#1 of the revised manuscript).*

*“Knowledge production used in this context refers to the ways scientific knowledge is produced and applied. Alternatively referred to as transdisciplinarity as it affords the integration of knowledge from academic and non-academic stakeholders (Zinsstag et al. 2009). Two modes of knowledge production are commonly characterised - mode 1 and 2. Whereas in mode 1 problems are set and solved in a context governed largely by the interest of a specific community/discipline, mode 2 knowledge is carried out in a context of application through collaborative working by multidisciplinary teams (Gibbon et al. 1994; Nowotny et al., 2001).”*

4. L102-4: Besides these pertinent aspects, the authors could consider mentioning that further aspects that require an integrated approach have been identified in the specific context of one health initiatives, namely improved equity, stewardship, and efficiency (<https://www.frontiersin.org/articles/10.3389/fpubh.2017.00020/full>)

*Response: Thank you for the comment. On page 5 (lines 106-108), we have added a sentence highlighting the added value of integrated approaches in the specific of context of One Health initiatives as suggested by the reviewer. The added sentence reads as follows:*

*“Besides, the integration of diverse knowledge on improved equity, stewardship and efficiency have been identified in the specific context of OneHealth initiatives (Ruegg et al. 2017).”*

5. L104-8: An important aspect that is missing here, is that citizens have tacit knowledge that must be mobilized for effective solutions. I.e. they have locally valid knowledge that is not scientifically recorded or available. In conventional approaches, such knowledge is often discovered throughout the project, but not included in the conception phase.

*Response: We agree that citizens have an important “stake” in the development of contextually-relevant and effective interventions. Indeed, in conceptualising and executing our MonkeyFeverRisk project (from which the present study is derived), we actively engaged with focal community groups in the KFD affected districts through household surveys and key informant interviews to understand their current and potential roles in developing contextually appropriate interventions for KFD. The results of this engagement has been reported elsewhere (see Asaaga et al. 2021a* [*https://doi.org/10.1371/journal.pntd.0009265*](https://doi.org/10.1371/journal.pntd.0009265)*).*

*These focal groups were not included in our framing for our co-production approach since the decision support tool was aimed at decision makers in health, animal health and environmental systems linked to KFD, who may wish to target their existing interventions at key at-risk groups or areas. Nonetheless, leveraging on our participatory engagement focal community groups (in respect of disease knowledge, occupational risk factors and information access pathways), we developed risk guidance translated into Kannada and Malayalam (the predominant local languages in the study districts) targeting affected communities as part of disease risk communication (see table 2 of the revised manuscript).*

6. L183: replace “actor” by “sector”.

*Response: Thank you for the comment. On page 9 (line 194) of the revised manuscript, we have replaced the word “actor” by “sector”.*

7. L206: The inclusion/exclusion of stakeholders in a co-production process has fundamental impact on its outcomes. Hence it would be important here to outline the stakeholder management plan, including the identification, prioritisation and engagement process.

*Response: Thank you for the comment. Please see our response to reviewer#3 point 4. On page 10 (lines 212-232) of the revised manuscript, we have added a new paragraph that further elucidates the stakeholder management plan, comprising the identification, prioritisation and engagement process.*

8. L214: From the affiliation mentioned above, it seems that no citizen representatives were engaged. This may seriously hamper the resulting conclusions in regard to their applicability.

*Response: Thank you for the comment. As explained in the methods section, the DST is essentially developed for frontline disease managers and not aimed at citizens. As such the non-participation of citizens in the multi-stakeholder workshops does not in any way hamper the applicability of our conclusions in this context as suggested by the reviewer. Suffice to say that as part of the overall project, we also conducted household surveys and key-informant interviews with households in affected communities, which afforded us the unique opportunity to understand contextual risk factors and disease-related impacts (for detail report on our household survey and key-informant interviews, see Asaaga et al. 2021a:* [*https://doi.org/10.1371/journal.pntd.0009265*](https://doi.org/10.1371/journal.pntd.0009265)*). Subsequent work on vulnerability, adaptation and contextual risk factors taking a more community centred approaches is currently underway in a related project.*

9. This should be discussed in the limitations of this study.
   L220: With “spillover”, usually events are described that are singular changes of host species, i.e. when SARS-CoV2 adapted from the intermediate host to humans, or NIPAH from pigs to humans. In the present case, the authors should refer to probability maps for human infections. A more detailed explanation of the differences can be found here: <https://www.frontiersin.org/articles/10.3389/fpubh.2020.596944/full>

*Response: The definition of spill-over events varies depending on the biology of the disease agent involved. We refer the reviewer to the papers by Sokolov et al. 2019 and Plowright et al. 2017 that highlight the series of hierarchical barriers that different types of pathogens have to overcome in order for spill-over to occur. As Sokolow et al. explain “Spill-over to humans can be common for some disease agents, as in the case of Lyme disease, where every human case is a spill-over event from a wildlife reservoir; or rare, as with HIV, which emerged after a handful of spill-over events of simian immunodeficiency virus mutated into HIV”. Since humans are dead-end hosts for Kyasanur Forest Disease, as for Lyme Disease, KFD falls into the category of disease for which spill-over events are common. We now clarify this point in the main text (please see footnote#2, page 7 of the revised manuscript).*

*“Aside from KFD affecting diverse forest users, it has a broad vector and host range, with a complex transmission cycle in which various tick species (notably the Haemaphysalis genus, but also Ixodes) and vertebrate hosts have been implicated, including wild rodents and shrews, monkeys and some birds (Pattnaik, 2006). Humans contract KFD when bitten by an infected tick but are incidental hosts for the disease and are not involved in onward transmission (Murhekar et al. 2015; Burthe et al. 2021). Therefore like the Lyme disease agent, Borrelia burgdorferi, KFD virus is a "spill-over pathogen" for which almost every human case represents a spill-over event from a wildlife reservoir via the infected tick vector (Burthe et al. 2021).”*

10. L223: It might be of interest to the readers, how the authors handled intellectual property rights at this point. "Allowing" implies that this would have been prohibited otherwise.

*Response: Thank you for the comment. On page 12 (line 275) of the revised manuscript, we have clarified that workshop participants were invited to use the DST and the engagement was experiential.*

11. L234: maybe state the objectives or provide a chart of the various steps in the co-production process with the corresponding stakeholders. E.g. it seems that the DST was developed in the lab by the authors, and then exposed to the assumed end-users for validation. It would be nice to see how the workshop1 information was integrated in the DST models.

*Response: Thank you for the comment. On page 20 (lines 399-409) of the revised manuscript, we have outlined how the workshop 1 information was integrated in the development of the DST. Please see below*:

*“Concerning the mapping and forecasting information needed to manage KFD, workshop participants were of the opinion that making available risk predictions at scales from village-level to clusters of villages would be most helpful to plan vaccination and awareness campaigns. Participants requested that data on environmental risk factors such as climate, land use change, altitude and livestock densities should appear alongside risk maps in a tool, together with contextual features like roads and household locations that health managers routinely use to plan their management across the landscape (Purse et al. 2020). Interfacing the seasonal activity of ticks with seasonal activities conducted by different groups of forest users in the tool was felt to be very important. Predictions of the month and villages at highest risk would be most useful at least six but better two months before the KFD season. The project team tailored the scale and appearance of the tools to these needs.”*

12. L240: consider revising the verb “inputted”

*Response:* Thank you for the comment. On page 14 (line 311) of the revised manuscript, we have replaced the word “inputted” by “entered” as suggested by the reviewer.

13. L241: I suggest revising this sentence: "The workshop relied entirely on participation using a range of different methods including group discussions, brainstorming and prioritization exercises." The "approach" refers to the "attitude of the researchers" or "the more fundamental paradigm", while what they are describing are participatory methods. Whether these are engaging or not (qualification of the participation) essentially depends on the facilitators, thus the authors would have to declare the criteria for their self-assessment to declare if participation was high.

*Response: Thank you for the comment. We agree that facilitators play in pivotal role in ensuring that the participation in the workshop discussions is engaging, and have revised the sentence to better articulate this point as suggested by the reviewer (see page 14, lines 312-314 of the revised manuscript). The added revised sentence now reads:“The facilitators adopted an opened-ended and friendly approach which ensured that the sessions were highly participatory, as participants were encouraged to feed in their knowledge and experience during the workshop using a range of different approaches including group discussions, brainstorming and prioritization exercises.”*

14. L244-6: What qualifies Fig 2 as a snapshot? As I understand, it is a graphical representation of the retrospective theory of change. Please omit “framework”. Please refine the process according to the comment above - or use a different figure.

*Response: Thank you for the comment. Based on the reviewer comment, we have revised figure 2 heading to read “retrospective theory of change on researcher expectations and the system-level outcomes of the co-production process adopted in the MonkeyFeverRisk project. Please see revised figure 2 below:*

***Fig.2.*** *Graphical representation of the retrospective theory of change on researcher expectations and the system-level outcomes of the co-production process adopted in the MonkeyFeverRisk project*. *The key components of the theory of change framework were as follows: (i) planned activities which included joint problem-framing with cross-sectoral actors to better understand the contextual risk factors and knowledge gaps, (ii) intermediate outputs comprising the social and ecological fieldwork, design and experimentation of a decision-support tool, and (iii) anticipated outcomes which ultimately contributes to fostering inter-sectoral collaboration and increased disease preparedness.*

15. L269: Consider using the heading "results". In my view, insights should be mentioned in the discussion section. In general please consider following the relevant guidelines from the equator network, e.g. the standards for reporting qualitative research:
    <https://www.equator-network.org/reporting-guidelines/srqr/>

*Response: Thank you for the suggestion. As explained above (in response to reviewer #5 comment on the structure of the manuscript), we have re-organised the entire manuscript including the results and discussion as suggested (see, for example, page 16 of the revised manuscript).*

16. L280: It is interesting to read that the modelling approach (spatial risk models) seems to have been pre-defined by the authors. Implicitly this excludes much information that is eminently important for early warning, such as the monkey deaths. I would like to read some reflection on the choice of model and its impact on the ability to respond to the stakeholder expectations. Alternatives would have been e.g. Fuzzy cognitive maps or agent based modelling. For a more comprehensive list of possible participatory modelling approaches please refer to Voinov, 2018: <https://doi.org/10.1016/j.envsoft.2018.08.028>

*Response: Thank you for the comment. Full justification for the choice of modelling methodologies and the reflections on the match with stakeholder knowledge and expectations can be found in the modelling paper by Purse et al. 2020:* [*https://doi.org/10.1371/journal.pntd.0008179*](https://doi.org/10.1371/journal.pntd.0008179)*. In brief, a statistical pattern matching approach to predicting spill-over geographically was selected since the empirical information on the ecological dynamics and human activities that lead to spill-over do not currently support application of more mechanistic approaches such as basic reproduction number or agent-based modelling approaches across landscapes. Machine learning approaches represent the state of the art for species distribution modelling so were the most scientifically robust methods to select for this application, given the lack of empirical knowledge on spill-over processes.*

17. L337: I assume, the authors mean "resolution" with “grain”.

*Response: Thank you for the comment. On page 33 (line 634) of the revised manuscript, we have replaced the word “grain” by “resolution”.*

18. L362: It is not quite clear to me, what ground-level means in this context and whether it is contrasted or synonym to the peripheral user, i.e. a field collaborator. Please specify the distinctions. It feels as if the authors were using a vertical and a concentric hierarchical image to express the same.

*Response: Apologies for the confusion. On page 25 (lines 448-449) of the revised manuscript, we have clarified that ‘ground-level’ users in this context refers to field health workers linked to primary health centres.*

19. L370: This rationale ignores the real time information about monkey deaths, which is highly relevant. The implemented algorithm endorses a heuristic approach, which doesn't necessarily reflect the true probability of infection, i.e. it is excessively conservative and identifies high probabilities, where they may not occur anymore, while they'd ignore evidence for high probabilities, where no human cases have occurred.
    Secondly the authors imply that the probability of infection equals the risk. However, in health sciences risk is usually the product of likelihood and consequences. In this case, the consequences would strongly depend on the subject being exposed to a certain likelihood. I suggest, the authors use “probability maps” or landscapes rather than "risk maps" as a term.

*Response: The information about monkey deaths is extremely sparse, covers a small fraction of the disease affected area and is spatially biased reflecting spatial variation in surveillance effort only so is not usable in a spatial risk model. The model is not intended or framed as estimating the probability of infection since there were no adequate data on infection rates of people, animal reservoirs and vectors or parameters on key infection processes to estimate this. The model, already peer-reviewed and published in PLoS NTDs, is framed as predicting the risk of spill-over to humans on the basis of the landscape conditions in which spill-over has occurred in the past, as indicated by human cases. Since we are keen to be clear that our maps focus on the probability of spill-over to humans, as opposed to the probability of infection or transmission, we do not agree that using “probability maps” gives more clarity than use of the term “risk maps” but we have now defined what we are using “risk maps” to mean more clearly in the text.*

20. L388: The authors could reflect whether the users they identified are actually stakeholders or not potentially shareholders in the problem. The distinction has nicely been elaborated in "Systemic Decision Making" by Hester and Adams (2017, Springer). Usually, the agents responsible for the management are not suffering from the consequences of poor management and consequently are share- rather than stakeholders in regard to their motivation(s) to act. This is quite relevant when it comes to the stewardship generated by integrative projects. The issue was illustrated by an evaluation of previous one health projects including one from Kerala on KFD: <https://www.frontiersin.org/articles/10.3389/fpubh.2021.653398/full>

*Response: Thanks for the suggestion. On page 26 (lines 478-480) of the revised manuscript, we have added a sentence that clarifies that the potential beneficiaries of the DST as identified by workshop participants are currently latent stakeholders who could potentially contribute to problem solving in respect of KFD prevention and control efforts. Please see the revised sentence below:*

*“This cohort of latent stakeholders were envisioned as having an important “stake” in contributing to holistic efforts to KFD prevention and control (cf. Hester & Adams, 2017: 139).”*

21. L401-4: This belongs in the methods

*Response: Thank you for the comment. Based on the reviewer’s comment, we have moved the related information on the experimentation workshop to the methods section (see page 12, lines 276-280 of the revised manuscript).*

22. L442 ff: The authors don't seem to be aware of the various efforts that have previously been made to learn from one health initiatives and other transdisciplinary work. A starting point would be the Hitziger 2021 paper, but multiple others have been published and should be used to compare the conclusions drawn by the authors here.
    In my view this discussion lacks references and reflection. While I acknowledge the self-comparison, I would highly recommend some external comparisons which would also allow to elaborate what was particularly exemplary in this project over other similar endeavors. e.g. <http://journals.sagepub.com/doi/10.1177/160940690700600401>; <http://link.springer.com/10.1007/s11625-015-0334-4>; <http://bmcpublichealth.biomedcentral.com/articles/10.1186/1471-2458-13-897>; <https://doi.org/10.1016/j.evalprogplan.2021.101991>; <https://www.frontiersin.org/research-topics/5479/>;

*Response: Thank you for the suggestion. As per our response to reviewer#5 general comment, we have restructured the entire manuscript to conform to the “standards for reporting qualitative research”. In particular, we have added a new discussion section to the revised manuscript, which reflects on findings of our study in the context of the wider OneHealth literature and the implications for future co-production of zoonotic disease tools and management (please see Section 4-discussion, lines 527-674, pages 29-35 of the revised manuscript)*

23. L466: Given the precedent project evaluated in Hitziger 2021, I don't think this claim “the first of its kind” is appropriate.

*Response: Thank you for the comment. Having reviewed Hitziger 2021, we found that the focal projects they evaluated in the Asian context did not encompass the instance reported in the present study. We have however revised the sentence to clarify that the present study is one of the first to use co-production approach to develop a decision support tool in the context of OneHealth at least in the Indian sub-continent context (please see line 585, page 31 of the revised manuscript).*

24. Figures: Fig2: Please also indicate how "culture of inter-sectorality, knowledge integration and preparedness were assessed (all other outcomes/ impacts have a criterion).

*Response:* Thank you for the comment. On page 14 we have revised figure 2 to clarify how culture of inter-sectorality, knowledge integration and preparedness were assessed as suggested by the reviewer. Please see the revised figure 2 below:

***Fig.2.*** *Graphical representation of the retrospective theory of change on researcher expectations and the system-level outcomes of the co-production process adopted in the MonkeyFeverRisk project*. *The key components of the theory of change framework were as follows: (i) planned activities which included joint problem-framing with cross-sectoral actors to better understand the contextual risk factors and knowledge gaps, (ii) intermediate outputs comprising the social and ecological fieldwork, design and experimentation of a decision-support tool, and (iii) anticipated outcomes which ultimately contributes to fostering inter-sectoral collaboration and increased disease preparedness.*

25. Fig3: please replace “spill-over” by “human infections”

*Response: Thank you the comment. We however respectfully disagree with the reviewer’s suggestion as we are not estimating probability of human infection through models.*

26. Tables: Table 1 and 2: please replace “spill-over” by “human infections”

*Response: Please see our response to reviewer #5 point 24. We feel that “spill-over” instead of “human infections” is most appropriate in this context as we are not estimating probability of human infections through models.*

*Thank you for the thorough and extremely valuable feedback on our manuscript.*
